# Associations of Multidomain Interventions With Improvements in Cognition in Mild Cognitive Impairment: A Systematic Review and Meta-analysis

**DOI:** 10.1001/jamanetworkopen.2022.6744

**Published:** 2022-05-03

**Authors:** Talia Salzman, Yanina Sarquis-Adamson, Surim Son, Manuel Montero-Odasso, Sarah Fraser

**Affiliations:** 1 Faculty of Health Sciences, School of Human Kinetics, University of Ottawa, Ontario, Canada; 2 Gait and Brain Lab, Parkwood Institute, Lawson Health Research Institute, London, Ontario, Canada; 3 Department of Epidemiology and Biostatistics, The University of Western Ontario, London, Ontario, Canada; 4 Schulich School of Medicine and Dentistry, Department of Medicine and Division of Geriatric Medicine, The University of Western Ontario, London, Ontario, Canada; 5 Faculty of Health Sciences, Interdisciplinary School of Health Sciences, University of Ottawa, Ontario, Canada

## Abstract

**Importance:**

Older adults with mild cognitive impairment (MCI) have the highest risk of progressing to dementia. Evidence suggests that nonpharmacological, single-domain interventions can prevent or delay progressive declines, but it is unclear whether greater cognitive benefits arise from multidomain interventions.

**Objective:**

To determine whether multidomain interventions, composed of 2 or more interventions, are associated with greater improvements in cognition among older adults with MCI than a single intervention on its own.

**Data Sources:**

MEDLINE, Embase, PsycInfo, AgeLine, CINAHL, and Cochrane Central Register of Controlled Trials were systematically searched from database inception to December 20, 2021.

**Study Selection:**

Included studies contained (1) an MCI diagnosis; (2) nonpharmacological, multidomain interventions that were compared with a single active control; (3) older adults aged 65 years and older; and (4) randomized clinical trials.

**Data Extraction and Synthesis:**

Data were screened and extracted by 3 independent reviewers. Following Preferred Reporting Items for Systematic Reviews and Meta-analyses (PRISMA) guidelines, random-effects meta-analyses were used to calculate effect sizes from the standardized mean difference (SMD) and 95% CIs.

**Main Outcomes and Measures:**

Postintervention cognitive test scores in 7 cognitive domains were compared between single-domain and multidomain groups. Exposure to the intervention was analyzed.

**Results:**

A total of 28 studies published between 2011 and 2021, including 2711 older adults with MCI, reported greater effect sizes in the multidomain group for global cognition (SMD, 0.41; 95% CI, 0.23-0.59; *P* < .001), executive function (SMD, 0.20; 95% CI, 0.04-0.36; *P* = .01), memory (SMD, 0.29; 95% CI, 0.14-0.45; *P* < .001), and verbal fluency (SMD, 0.30; 95% CI, 0.12-0.49; *P* = .001). The Mini-Mental State Examination (SMD, 0.40; 95% CI, 0.17-0.64; *P* < .001), category verbal fluency test (SMD, 0.34; 95% CI, 0.13-0.56; *P* = .002), Trail Making Test–B (SMD, 0.46; 95% CI, 0.13-0.80; *P* = .007), and Wechsler Memory Scale–Logical Memory I (SMD, 0.47; 95% CI, 0.15-0.80; *P* < .001) and II (SMD, 0.26; 95% CI, 0.07-0.45; *P* < .001) favored the multidomain group. Exposure to the intervention varied between studies: the mean (SD) duration was 71.3 (36.0) minutes for 19.8 (14.6) weeks with sessions taking place 2.5 (1.1) times per week, and all interventions lasted less than 1 year.

**Conclusions and Relevance:**

In this study, short-term multidomain interventions (<1 year) were associated with improvements in global cognition, executive function, memory, and verbal fluency compared with single interventions in older adults with MCI.

## Key Points

**Question:**

Are multidomain interventions associated with better cognitive outcomes than single interventions in older adults with mild cognitive impairment (MCI)?

**Findings:**

This meta-analysis of 28 studies with 2711 participants examined global cognition, attention, executive function, memory, processing speed, and verbal fluency effects. After intervention, significant improvements favoring multidomain interventions were observed in global cognition, executive function, memory, and verbal fluency compared with the single-intervention, active control.

**Meaning:**

In this study, multidomain interventions were more strongly associated with improving global cognition, memory, executive function, and verbal fluency in older adults with MCI than single interventions, but evidence is needed to determine the optimal length of multidomain interventions.

## Teaser

This systematic review and meta-analysis evaluates whether multidomain interventions, composed of 2 or more interventions, are associated with greater improvements in cognition among older adults with mild cognitive impairment (MCI) than a single intervention on its own.

## Introduction

Mild cognitive impairment (MCI) refers to an intermediate stage between normal aging and dementia in which individuals report cognitive concerns and demonstrate objective cognitive deficits that do not interfere with daily functioning.^1^ Recent evidence has demonstrated that the prevalence of MCI increases from 6.7% in older adults aged 60 to 64 years to 25.2% in those aged 80 to 84 years.^2^ Although MCI may be considered a prodromal stage of dementia,^2^ 35% of older adults with MCI revert back to a normal cognitive state.^3,4^

Nonpharmacological interventions, including cognitive training, can help improve one’s mood and preserve cognitive functions such as memory.^5,6^ Exercise can also lead to elevated cerebral metabolism and brain-derived neurotrophic factor (BDNF), which support brain plasticity and angiogenesis in the hippocampus.^7,8,9^ Multidomain interventions, composed of 2 or more interventions, may have even greater benefits than cognitive training or exercise alone.^10,11,12^ The effects of multidomain interventions have been examined in healthy older adults.^13,14,15^ However, there is inconclusive evidence to support significantly improved cognitive outcomes following multidomain vs single-domain interventions in MCI.^16,17^ Additionally, reviews on this topic have primarily focused on memory or are limited to investigations of cognitive and physical training, while interventions such as mindfulness and nutrition are overlooked.^17,18,19^

The purpose of this systematic review and meta-analysis is to compare cognitive outcomes immediately following nonpharmacological single-domain and multidomain interventions in older adults with MCI. This article (1) examines whether multidomain interventions are associated with greater improvements in cognition than single-domain interventions; (2) evaluates which cognitive domains are associated with improvements following multidomain interventions; and (3) assesses length of exposure to the multidomain intervention that is associated with positive outcomes in MCI. Understanding the benefits of multidomain interventions will help design more robust intervention strategies that preserve cognition or delay the onset of decline in older adults with MCI.

## Methods

This review has been registered in the Prospective Register of Systematic Reviews (CRD42019126899). Results followed the Preferred Reporting Items for Systematic Reviews and Meta-Analyses (PRISMA) reporting guideline.^20^

### Eligibility Criteria

Inclusion and exclusion criteria were outlined using the PICO (population, intervention, comparison, and outcome) model.^21^ Eligible studies were randomized clinical trials that examined older adults aged 65 years or older who have been diagnosed with MCI using Petersen criteria and confirmation from clinicians who assessed subjective cognitive concerns, objective deficits compared with age-related norms, no functional deficits related to cognition, and the absence of dementia.^1^ Interventions were considered multidomain if they included 2 or more nonpharmacological components (eg, cognitive and aerobic exercise) that were completed simultaneously or sequentially.^7^ Additionally, studies had to compare the multidomain intervention with an active control (ie, a single intervention). Systematic reviews, studies without full texts, interventions with a pharmacological component, and studies comparing multidomain interventions with inactive controls (ie, waiting list controls) were excluded. Additionally, studies whose primary aim was to examine the effects of multidomain interventions on MCI and a cooccurring condition that can affect cognition (eg, MCI and a neurological disorder) were excluded.

### Search Strategy

The literature search was conducted in December 2021 in MEDLINE, Embase, AgeLine, PsycInfo, CINAHL, and Cochrane Central Register of Controlled Trials (CENTRAL) with no restrictions on publication date or language. The search strategy combined medical subject heading terms and keywords encompassing 4 key concepts: cognitive status, age, intervention, and study design (eTable in the Supplement).

Studies were independently screened by 3 of us (S.F., S.S., and T.S.) in 2 steps: (1) title and abstract screening and (2) full-text screening. Relevant abstracts were retained for the full-text review based on the inclusion and exclusion criteria. Study and participant characteristics and outcome measures were extracted from each study by 3 of us (S.F., S.S., and T.S.). During the screening and extraction stages, disagreements were resolved by consensus between authors.

### Risk of Bias

Risk of bias (RoB) was assessed by 3 of us (S.F., S.S., and T.S.) using the Cochrane RoB tool for randomized controlled studies that evaluated selection, performance, detection, attrition, reporting, and other sources of bias.^22^ Each criterion was classified according to a low, high, or unclear risk of bias. Disagreements within these categories were resolved by consensus between authors. The Egger test of funnel plot asymmetry was used to visualize publication bias.^23^

### Statistical Analysis

Cognitive outcomes were analyzed by cognitive domain (eg, executive function), and meta-analyses were conducted when there were at least 3 studies for a given outcome. A random-effects model was used to account for methodological differences and between-study variance. Review Manager version 5.4 was used to calculate Hedge *g* and pooled effect size estimates.^24^ Hedge *g* was derived directly from the published data, and means and SDs were requested from the corresponding authors when they were not reported.^25^ Differences between single-domain and multidomain interventions were quantified as the change between scores at baseline and immediately after the intervention. The standardized mean difference (SMD) was calculated by pooling SDs for pre-post change scores and pooling posttest SDs for posttest scores.^26^ The primary outcome was reported when studies used multiple measures for the same cognitive domain, and the comparison of the marginal analyses was used to assess factorial trials.^27^ Mean scores were multiplied by −1 to ensure higher scores favored improvements in the multidomain group.^28^ Effect sizes were then interpreted using small (*d* = 0.20), medium (*d* = 0.50), and large (*d* = 0.80) effect size categories.^29^ For all analyses, a 2-tailed *P* < .05 was considered statistically significant. Exposure to the intervention was assessed by the number of sessions, duration, and frequency.

The *I*^2^ statistic was used to evaluate potential sources of heterogeneity and was classified into small (≤25%), medium (26-74%), and large (≥75%) groups.^30^ Subgroup analyses were conducted when there was medium or large heterogeneity across studies. When sufficient studies were available, subgroups were created based on 5 categories: (1) recruitment source (ie, community, clinic-based); (2) multidomain intervention type (eg, cognitive-physical); (3) single-intervention type (ie, a single component of the multidomain intervention or an alternate intervention); (4) multidomain intervention style (ie, group, individual); and (5) order of the multidomain intervention components (ie, sequential, simultaneous). Additionally, specific cognitive tests (eg, Mini-Mental State Examination [MMSE]) were analyzed within each cognitive domain.

## Results

### Study Selection

**Figure 1.**
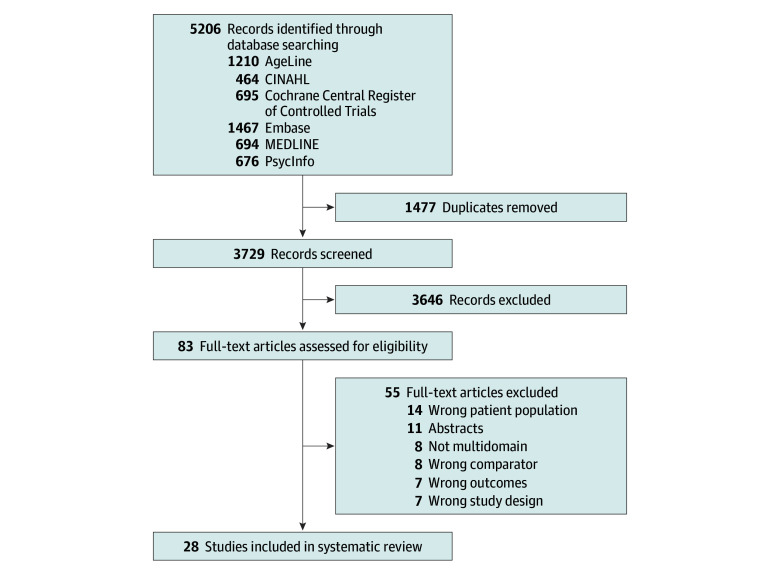
Flow Diagram of Study Selection

Database searches in MEDLINE, Embase, PsycInfo, CINAHL, AgeLine, and CENTRAL yielded 5206 studies that were managed in Covidence. Titles and abstracts were screened following the removal of 1477 duplicates, and 83 studies remained for the full-text review. Fifty-five studies were then excluded for having an abstract only, wrong patient population, no multidomain intervention, wrong comparator, wrong outcome, or wrong study design (Figure 1). Finally, 28 studies, with 2711 participants, were retained for the meta-analyses.

### Study Characteristics

The publication period ranged from 2011 to 2021, as summarized in Table 1.^31,32,33,34,35,36,37,38,39,40,41,42,43,44,45,46,47,48,49,50,51,52,53,54,55,56,57,58^ Seventeen studies recruited participants from the community^31,32,36,37,39,40,48,49,50,51,52,53,54,55,56,57,58^ whereas 11 recruited from memory clinics.^33,34,35,38,41,42,43,44,45,46,47^ Total sample sizes ranged from 22 to 555, with a mean (SD) age of 71.6 (3.4) years. MCI was diagnosed using a combination of Petersen criteria, MMSE, Montreal Cognitive Assessment (MoCA), clinical dementia rating (CDR) scores, and evaluations by physicians.

**Table 1.** Participant and Study Characteristics of Included Studies

Source	Country	Recruitment source	Sample size, No.	Age, mean (SD), y	Sex, % female	MCI diagnosis criteria	Baseline cognition, mean (SD)	Intervention and duration	Time points	Multidomain cognitive outcome
Bae et al,^31^ 2019	Japan	Community	Multidomain intervention: 41	75.5 (6.0)	43.9	Objective cognitive impairment (NCGG-FAT); MMSE ≥24; functional independence; no dementia	MMSE: 27.1 (2.1)	Multidomain intervention: “KENKOJISEICHI” physical activities (body movement or strength), cognitive activities (mental engagement), and social activities (socializing in the community) for 90 min, 2/wk over 24 wk (48 sessions), sequential. Active control: health education classes on oral care and nutrition; two 90-min sessions over 24 wk. Interventions supervised in-person by trained nonhealth professionals.	Baseline and 24 wk	↑ Spatial working memory (*P* = .02) in multidomain intervention group compared with active control after 24 wk.
			Active control: 42	76.4 (5.1)	52.4		MMSE: 26.7 (2.0)			
Bai et al,^32^ 2021	China	Community	Multidomain intervention: 34	66.7 (5.8)	59	Petersen criteria	MMSE: 20.2 (3.2)	Multidomain intervention: 0.8 mg folic acid/d and 2 capsules of 800 mg DHA/d, simultaneous. Folic acid: 0.8 mg folic acid and 2 soybean oil capsules per day. DHA: 2 capsules 800 mg DHA and 1 cornstarch pill per day. Other control: 1 cornstarch pill and 2 soybean oil capsules per day. Interventions supervised in-person by researchers and physicians.	Baseline, 26 wk, 52 wk follow-up	↑ Arithmetic (*P* = .009), block design (*P* = .006), and picture arrangement (*P* = .046) scores on WAIS subtests in multidomain group compared with folic acid group at 6 mo. No differences in information, digit span, and picture completion.
			Folic acid: 35	67.5 (5.1)	66		MMSE: 20.4 (2.4)			
			DHA: 36	70.2 (6.5)	70		MMSE: 20.5 (2.6)			
			Other control: 33	68.3 (6.4)	58		MMSE: 21.3 (2.2)			
Bisbe et al,^33^ 2020	Spain	Clinic	Multidomain intervention: 14	77.3 (5.2)	50	aMCI: Petersen criteria; MMSE score ≥24; CDR, 0.5	MMSE: 27.4 (2.1)	Multidomain intervention: strength, endurance, flexibility, balance, coordination, and gait training for 60 min, 2/wk for 12 wk, sequential. Active control: choreographed aerobic dance sessions that involved learning the steps with instructions from a physical therapist, performing the choreography with a video tutorial, and dancing with music only for 60 min, 2/wk for 12 wk. Interventions supervised in-person by physiotherapists.	Baseline and 12 wk	↑ WMS-LM III verbal recognition in active control compared with multidomain group (*P* = .003) after 12 wk. No differences in RBANS scores.
			Active control: 17	72.9 (5.6)	52.9		MMSE: 27.3 (1.9)			
Buschert et al,^34^ 2011	Germany	Clinic	Multidomain intervention: 12	71.8 (8.6)	50	aMCI: Petersen criteria; MMSE >22	ADAS-Cog: 8.7 (2.9); MMSE: 28.1 (1.5)	Multidomain intervention: cognitive training (ie, teaching and practicing theoretically motivated strategies and skills to optimize cognitive functioning) and stimulation (ie, engagement in a range of activities and discussions aimed at general enhancement of cognitive and social functioning) for 120 min, 1/wk for 6 mo, sequential. Active control: paper-pencil exercises for self-study that focused on sustained attention for 60 min 1/mo for 6 mo. Intervention supervised in-person by instructors.	Baseline, 26 wk	↑ ADAS-Cog (*P* = .02) performance in multidomain intervention compared with the control. Differences in MMSE scores were not significant (*P* = .07).
			Active control: 12	70.7 (5.7)	50		ADAS-Cog: 9.8 (4.3); MMSE:26.8 (1.5)			
Combourieu Donnezan et al,^35^ 2018^a^	France	Clinic	Multidomain intervention: 21	77.1 (1.4)	NR	Single and multiple domain MCI: Petersen criteria	28.1 (0.36)	Multidomain intervention: HAPPYneuron cognitive gaming software (attention, executive functions, working memory, mental flexibility, inhibition, reasoning and updating) and aerobic training on bikes for 1 h, 2/wk for 12 wk (24 sessions), simultaneous. Cognitive control: HAPPYneuron cognitive gaming software (attention, executive functions, working memory, mental flexibility, inhibition, reasoning and updating) for 1 h, 2/wk for 12 wk (24 sessions). Exercise control: aerobic training on bikes for 1 h, 2/wk for 12 wk (24 sessions). Inactive control: no intervention. Intervention supervised in-person by physiotherapists.	Baseline, 12-wk and 38-wk follow-up	No significant group differences in executive functions between single and multidomain interventions.
			Cognitive control: 19	76.3 (1.5)	NR		MMSE: 27.3 (0.42)			
			Exercise control: 21	75.2 (1.3)	NR		MMSE: 28.2 (0.43)			
			Inactive control: 15	79.2 (4.0)	NR		MMSE: 27.3 (0.50)			
Damirchi et al,^36^ 2018	Iran	Community	Multidomain intervention: 13	67.8 (4.7)	100	MMSE score and examination by a neurologist	MMSE: 23.3 (1.8)	Multidomain intervention: combined physical and mental training for 30-60 min/wk of mental training and 11-45 min 3/wk physical training for 8 wk, sequential. Exercise control: aerobic and muscular strength and range of movement training for 11-45 min, 3/wk for 8 wk. Mental control: Modified My Better Mind computer program that trained attention, working memory, processing speed, executive processing, visual-spatial and spatial executive processing for 30 min, doubled at 7-8 wk. Inactive control: no intervention. Intervention supervised in-person by physiotherapists.	Baseline, 8 wk and 26 wk follow-up	No differences in working memory, processing speed, and Stroop reaction time and errors between multidomain intervention group and control groups.
			Exercise control: 11	68.8 (3.7)	100		MMSE: 23.2 (2.2)			
			Mental control:: 11	67.9 (3.8)	100		MMSE: 23.8 (2.0)			
			Inactive control: 9	69.1 (5.0)	100		MMSE: 23.4 (2.1)			
Fiatarone Singh et al,^37^ 2014	Australia	Community	Multidomain intervention: 27	70.1 (6.7) y^b^	68^b^	Petersen criteria	ADAS-Cog: 7.8 (4.2); MMSE: 27.0 (2.0)	Multidomain intervention: exercise (high intensity progressive resistance training) and computerized multidomain cognitive training (COGPACK verbal memory, executive function, attention, and processing speed) for 100 min, 2-3/wk for 6 mo, sequential. Cognitive control: computerized multidomain cognitive training (COGPACK verbal memory, executive function, attention, and processing speed) for 75 min, 2-3/wk for 6 mo. Exercise control: exercise (high intensity progressive resistance training) for 75 min, 2-3/wk for 6 mo. Active control: watching videos/quizzes and seated calisthenics for 60 min, 2-3/wk for 6 mo. Interventions supervised in-person by research assistants from exercise physiology or with physical therapy backgrounds.	Baseline, 6-mo and 18-mo follow-up	↑ Executive function in exercise control compared to multidomain intervention group (*P* < .03) at 6 and 18 mo (*P* = .02). ↑ Global cognition in exercise control compared with multidomain intervention group at 18 mo (*P* < .04).
			Cognitive control: 24				ADAS-Cog: 8.1 (3.8); MMSE: 28.0 (2.0)			
			Exercise control: 22				ADAS-Cog: 7.9 (2.8); MMSE: 27.0 (1.0)			
			Active control: 27				ADAS-Cog: 7.9 (3.1); MMSE: 27.0 (2.0)			
Fogarty et al,^38^ 2016	Canada	Clinic	Multidomain intervention: 26	71.6 (9.3)	50	Single and multiple domain aMCI: Petersen criteria; interview with participant and informant; medical history	MMSE: 28.3 (1.4); MoCA, 23.7 (1.8)	Multidomain intervention: Taoist Tai Chi and memory intervention program that teaches about memory strategies and lifestyle factors that impact memory for 90 min, 2/wk for 10 wk (20 sessions), sequential. Cognitive control: memory intervention program for 6 sessions with 2 follow-ups at 1 and 3 mo. Intervention supervised in-person by Tai Chi instructor.	Baseline, after 10 and 22 wk	No significant group differences between single and multidomain interventions.
			Cognitive control: 22	72.6 (5.8)	55		MMSE: 27.9 (1.1); MoCA: 24.8 (2.0)			
Gill et al,^39^ 2016	Canada	Community	Intervention: 22	72.6 (7.4)	15	MoCA score <27; MMSE score <24; physician diagnosis	MMSE: 28.7 (1.0); MoCA: 25.1 (2.1)	Multidomain intervention: square stepping exercise, exercise (aerobic, strength, balance, and flexibility training) while responding to semantic and phonemic verbal fluency tasks, and randomly generated arithmetic (dual-task) for 50-75 min dual-task and 45 min square stepping exercise 2-3/wk for 26 wk, simultaneous. Exercise control: square stepping exercise and exercise (aerobic, strength, balance, and flexibility training) for 50-75 min exercise and 45 min square stepping 2-3/wk for 26 wk. Intervention supervised in-person by fitness instructors.	Baseline, 12, 26, and 52 wk	↑ Composite global cognition (executive function, processing speed, verbal memory, verbal fluency; (*P* = .04); ↑ verbal memory (*P* = .02) and ↑ verbal fluency (*P* = .003) in multidomain intervention group compared with exercise control at 26 wk.
			Exercise control: 20	74.5 (7.0)	15		MMSE: 28.9 (1.3); MoCA: 24.7 (1.7)			
Greblo Jurakic et al,^40^ 2017	Croatia	Community	Multidomain intervention: 14	69.4 (4.1)	100	MoCA score 19-25.	MoCA: 23.4 (1.7)	Multidomain intervention: balance and core resistance training (ie, HUBER; push and pull exercises on the handles in different postures, hand positions, and directions) for 30 min, 3/wk for 8 wk, sequential. Active control: Pilates (ie, supine, side-lying, sitting, and quadruped exercises) for 60 min, 3/wk for 8 wk. Intervention supervision not reported.	Baseline, 8 wk.	↑ Overall MoCA score (*P* < .05), MoCA subtests of visuospatial/ executive (*P* < .05), and attention (*P* < .05) in multidomain group compared with control.
			Active control: 14	71.4 (3.7)	100		MMSE: 25.8 (1.5)			
Hagovská et al,^41^ 2016	Slovakia	Clinic	Intervention: 40	68.2 (6.7)	45	Confirmed by psychiatrist or psychologist (*ICD-9-CM code* 331.83). Exclusion: MMSE score ≤23.	MMSE: 26.0 (2.6)	Multidomain intervention: CogniPlus training battery (attention, working memory, long-term memory, executive functions, visuomotor coordination, and spatial processing) and motor training (walking under different conditions) for 30 min, 2/wk for 10 wk (20 sessions), sequential. Exercise control: balance training (walking under different conditions) for 30 min/d for 10 wk. Intervention supervised in-person by physiotherapists.	Baseline and after 10-wk intervention	There were 5 significant correlations in the multidomain intervention group between balance control, cognitive functions, gait speed, and activities of daily living compared with 1 in exercise control between balance control and gait speed at 10 wk.
			Exercise control: 40	65.7 (5.6)	52		MMSE: 26.0 (1.5)			
Hagovská et al,^42^ 2016	Slovakia	Clinic	Intervention: 40	68.0 (4.4)	55	Confirmed by psychiatrist or psychologist (*ICD-9-CM* code 331.83). Exclusion: MMSE score ≤23	MMSE: 25.9 (7.3)	Multidomain intervention: CogniPlus training battery (attention, long-term memory, executive functions, working memory, visual-motor coordination) for 30 min 2/wk for 10 wk (20 sessions), sequential. Intervention supervised in-person by physiotherapists. Exercise control: balance training (walking under different conditions) for 30 min/d for 10 wk.	Baseline and after 10-wk intervention	↑ Overall Addenbrooke’s cognitive examination (*P* = .03) and ↑ in subdomains of attention (*P* = .002), memory (*P* = .007), and language (*P* < .001) after 10 wk in multidomain intervention group compared with exercise control.
			Exercise control: 40	65.9 (6.2)	48		MMSE: 26.8 (6.8)			
Hagovská et al,^43^ 2016	Slovakia	Clinic	Intervention: 40	68.0 (4.4)	45	Confirmed by psychiatrist or psychologist (*ICD-9-*CM 331.83). Exclusion: MMSE score ≤23	MMSE: 26.0 (2.6)	Multidomain intervention: CogniPlus training battery (attention, working memory, long-term memory, executive functions, visuomotor coordination, and spatial processing) and motor training (walking under different conditions) for 30 min, 2/wk for 10 wk (20 sessions), sequential. Exercise control: balance training (walking under different conditions) for 30 min/d for 10 wk. Intervention supervised in-person by physiotherapists.	Baseline and after 10-wk intervention	↑ MMSE score in multidomain intervention group compared to exercise control after 10 wk (*P* = .04).
			Exercise control: 40	65.9 (6.2)	52		MMSE: 26.0 (1.5)			
Hagovská et al,^44^ 2016	Slovakia	Clinic	Multidomain intervention: 40	68.0 (4.4)	55	Confirmed by psychiatrist or psychologist (*ICD-9-CM* code 331.83). Exclusion: MMSE score ≤23	MMSE: 26.0 (2.6)	Multidomain intervention: CogniPlus training battery (attention, long-term memory, executive functions, working memory, visual-motor coordination) for 30 min, 2/wk for 10 wk (20 sessions), sequential. Exercise control: balance training (walking under different conditions) for 30 min/d for 10 wk. Intervention supervised in-person by training staff.	Baseline and after 10-wk intervention	↑ Performance on MMSE (*P* < .001), AVLT (*P* < .001), Stroop (*P* < .002), DRT (*P* < .005), and TMT-A (*P* < .01) in multidomain intervention group compared with exercise control.
			Exercise control: 40	65.9 (6.2)	48		MMSE: 26.0 (1.5)			
Jeong et al,^45^ 2021	South Korea	Clinic	Multidomain intervention: 13	70.2 (7.5)	69	Petersen criteria and clinical interview and neurological examination	MMSE: 25.8 (2.3); ADAS-Cog: 24.1 (7.8)	Multidomain intervention: aerobic and cognitive training (eg, counting, word games, simple memory span) and health education classes for 90 min, 2/wk for 12 wk, simultaneous. Active control: monthly health education classes (3 sessions). Intervention supervised in-person by 2 geriatric exercise specialists and 1 occupational therapist or nurse.	Baseline, 12 wk	No differences in MMSE (*P* = .72) or ADAS-Cog (*P* = .11) between multidomain intervention and control. Improvements in TMT-A (*P* < .05), TMT-B (*P* = .01), and DSST (*P* = .02) in multidomain group compared with control.
			Active control: 13	71.8 (5.5)	69		MMSE: 25.0 (2.6); ADAS-Cog: 28.5 (8.4)			
Kim et al,^46^ 2020	South Korea	Clinic	Multidomain intervention: 16	70.0 (6.0)	87.5	Petersen criteria, MMSE score 20-23; MoCA score 0-22.	ADAS-Cog: 11.1 (4.1); MoCA: 18.8 (2.5)	Multidomain intervention: electroacupuncture and computer-based cognitive rehabilitation (eg, attention, memory, and executive functions) for 30 min, 3/wk for 8 wk for each component, sequential. Active control: electroacupuncture 30-min sessions, 3/wk, for 8 wk (24 sessions). Intervention supervised in-person by physicians.	Baseline, 8 wk, 20 wk.	No significant differences in ADAS-Cog scores between groups at 8 or 20 mo.
			Active control: 16	74.3 (5.4)	87.5		ADAS-Cog: 11.2 (6.2); MoCA: 19.3 (3.0)			
Köbe et al,^47^ 2016	Germany	Clinic	Multidomain intervention: 13	70.0 (7.2)	31	Single and multiple domain MCI: Petersen criteria	MMSE: 28.5 (1.1)	Multidomain intervention: cognitive stimulation (AKTIVA cognitively stimulating leisure activities and memory strategies) started wk 4 with 1 individual and 12 group 90-min sessions; aerobic training (cycle ergometer) for 45 min 2/wk for 6 mo, omega-3 FA (2.2 g/d) every day for 6 mo, sequential. Active control: nonaerobic training (stretching and toning) for 45 min 2/wk for 6 mo and omega-3 FA (2.2 g/d) every day over 6 mo. Intervention supervised in-person by trained exercise leaders.	Baseline and after 24-wk intervention	No significant differences in cognitive performance between intervention groups.
			Active control: 9	70.0 (5.2)	44		MMSE: 27.9 (1.7)			
Lam et al,^48^ 2015	China	Community	Multidomain intervention: 132	76.3 (6.6)	78	Single and multiple domain MCI: Subjective concerns, objective measures of episodic memory, verbal fluency, and attention. CDR ≥1 exclusion	ADAS-cog: 11.6 (3.4); MMSE: 25.2 (2.2)	Multidomain intervention: integrated 1 cognitive and 2 types of mind-body exercises for 1 h, 3/wk for 12 mo, sequential. Cognitive control: cognitively demanding activities (reading and discussing newspapers, playing board games) for 1 h 3/wk for 12 mo. Exercise control: physical exercise (stretching and toning exercise, Tai Chi, and 1 aerobic exercise) for 1 h, 3/wk for 12 mo. Active control: social activity (tea gathering, film watching) for 1 h, 3/wk for 12 mo. Intervention supervised in-person by staff members at the social centers and at home by family members.	Baseline, 12, 32 and 52 wk	No significant differences in cognitive outcomes (CDR-SOB, Chinese MMSE) between intervention groups.
			Cognitive control: 145	74.4 (6.4)	79		ADAS-Cog: 11.3 (3.2); MMSE: 25.7 (2.4)			
			Exercise control: 147	75.7 (6.7)	77		ADAS-Cog: 11.7 (3.3); MMSE: 25.8 (2.3)			
			Active control: 131	75.4 (6.1)	78		ADAS-Cog: 11.5 (3.4); MMSE: 25.6 (2.4)			
Li et al,^49^ 2021^c^	China	Community	Multidomain intervention: 42	NR	64.3	Petersen criteria	MMSE: 26.5 (1.3); MoCA: 21.5 (2.1)	Multidomain intervention: aerobic, strength, balance, coordination, and sensitivity exercise training for 30 min, 5/wk for 6 mo, sequential. Active control: community health instruction for 1 h/mo. Intervention supervised in-person by two research member instructors.	Baseline, 12, and 26 wk	↑ MMSE (*P* < .001) and MoCA (*P* < .001) scores in multidomain group compared with control at 3 and 6 mo.
			Active control: 42	NR	57.1		MMSE: 26.6 (1.5); MoCA: 21.1 (2.0)			
Makizako et al,^50^ 2012	Japan	Community	Multidomain intervention: 25	75.3 (7.5)	48	aMCI: Petersen criteria, MMSE score 24-30, CDR = 0.5.	MMSE: 26.8 (1.8)	Multidomain intervention: aerobic exercises, muscle strength training, postural balance retraining, and combined dual-task exercises (cognitive task with exercise) for 90 min over 6 mo (40 sessions), simultaneous. Active control: health promotion classes. Two classes over 6 mo. Interventions supervised in-person by two trained physiotherapists.	Baseline and after 24 wk.	Reaction times and dual-task costs were not significantly different between multidomain intervention group and active control after 6-mo intervention.
			Active control: 25	76.8 (6.8)	44		MMSE: 26.6 (1.6)			
Martin et al,^51^ 2019	Australia	Community	Multidomain intervention: 33	71.8 (6.4)	61	Single and multiple domain MCI: Petersen criteria, RBANS, WTAR, BADL.	MMSE: 26.8 (1.8)	Multidomain intervention: active tDCS and COGPACK cognitive training for learning and memory; 45-60 min cognitive training with active tDCS (2 mA for 30 min and 0.016 mA for 30 min) 3/wk over 5 wk (15 sessions total), simultaneous. Active control: sham tDCS (0.016 mA for 60 min) 3/wk over 5 wk (15 sessions total). Interventions supervised in-person by researchers.	Baseline, 5 wk, and 12 wk follow-up.	No significant differences in verbal memory between the multidomain intervention and control groups.
			Active control: 35	71.6 (6.4)	71		MMSE: 26.6 (1.6)			
Shimada et al,^52^ 2018	Japan	Community	Multidomain intervention: 154	71.6 (5.0)	50	aMCI and naMCI: Petersen criteria.	MMSE:26.6 (1.8)	Multidomain intervention: “Cognicize” dual-task training including physical (aerobic exercise, muscle strength training, postural balance retraining) and cognitive tasks for 90 min, 1/wk for 40 wk, simultaneous. Active control: health promotion classes on aging, nutrition, oral care, frailty, and urinary incontinence; 3 health promotion classes (90 min each) over 40 wk. Intervention supervised in-person by geriatric physiotherapists and 5 instructors at a fitness facility.	Baseline and after 40-wk intervention.	↑ MMSE (*P* = .012), WMS-LM II (*P* = .004), verbal fluency letter (*P* < .001), and category (*P* = .002) scores in multidomain intervention group compared with active control after 40-wk intervention. No differences in RAVLT (*P* = .35).
			Active control: 154	71.6 (4.9)	50		MMSE: 26.8 (1.8)			
Shimizu et al,^53^ 2018	Japan	Community	Multidomain intervention: 34	74.9 (4.3)	82	MCI: Petersen criteria.	FAB: 15.0 (2.0)	Multidomain intervention: instructor-led exercises with rhythmic background music and Naruko clapper for 30 min, 1/wk for 12 wk, simultaneous. Active control: instructor-led exercises without Naruko clapper for 30 min, 1/wk for 12 wk. Intervention supervised in-person by researchers and staff at public health centers.	Baseline and after 12-wk intervention.	No significant differences in cognitive performance between intervention groups.
			Active control: 10	73.3 (7.3)	91		FAB: 15.1 (2.0)			
Sun et al,^54^ 2021	China	Community	Multidomain intervention: 37	Age for whole sample: 70.83 (6.54).	56.8	MCI and aMCI: Petersen criteria.	MMSE: 26.68 (1.90); MoCA: 21.11 (2.22)	Multidomain intervention: acupressure and cognitive training for 60 min, 5/wk for 6 mo for both interventions, sequential. Cognitive control: cognitive training in attention, memory, calculation, language, and executive functions for 60 min 5x/wk for 6 mo. Acupressure control: based on acupuncture on Baihui (GV20), Fengchi (GB20), Shenting (GV24), Sishencong (EX- HN1) and Taiyang (EX-HN5) acupoints; 20 min/session, 2-3 sessions/d, 5/wk for 6 mo. Education control: health education lectures; 1/mo for 60 min (6 sessions). Intervention supervised in-person by research assistant and group leaders and at home by family members.	Baseline, 3, and 24 wk.	↑ MMSE in multidomain intervention group compared with acupressure (*P* = .03) and cognitive training (*P* = .03) groups. ↑ MoCA in multidomain intervention group compared with acupressure (*P* = .002) and cognitive training (*P* < .007) groups.
			Cognitive control: 38		52.6		MMSE: 26.71 (1.68); MoCA: 21.53 (2.00)			
			Acupressure control: 38		73.7		MMSE: 26.87 (1.74): MoCA: 21.47 (2.22)			
			Education control: 38		73.7		MMSE: 26.37 (1.82); MoCA: 21.05 (2.50)			
Suzuki et al,^55^ 2013	Japan	Community	Multidomain intervention: 50	74.8 (7.4).	50	MCI and aMCI: Petersen criteria.	ADAS-cog: 6.0 (2.8); MMSE: 26.8 (2.3)	Multidomain intervention: aerobic exercises, muscle strength training, postural balance retraining, and combined dual-task exercises (cognitive task with exercise) for 90 min, 2/wk, over 12 mo (80 sessions), simultaneous. Active control: health promotion classes; 3 classes over 12 mo. Intervention supervised in-person by 2 trained physiotherapists involved in geriatric rehabilitation.	Baseline, after 24, and 52 wk.	↑ MMSE score (*P* = .04) and WMS-LM I immediate recall (*P* = .04) at 6 mo in multidomain intervention group compared with the control. No differences in WMS-LM II and ADAS-Cog between groups.
			Active control: 50	75.8 (6.1)	48		ADAS-Cog: 6.5 (2.8); MMSE: 26.3 (2.7)			
Suzuki et al,^56^ 2012	Japan	Community	Multidomain intervention: 25	75.3 (7.5)	48	aMCI: Petersen criteria.	MMSE: 26.8 (1.8)	Multidomain intervention: aerobic exercises, muscle strength training, postural balance retraining, and combined dual-task exercises (cognitive task with exercise) for 90 min, 2/wk, over 6 mo (40 sessions), simultaneous. Active control: health promotion classes; 2 classes over 6 mo. Intervention supervised in-person by 2 physiotherapists involved in geriatric rehabilitation and 3 well-trained instructors.	Baseline and after 24 wk.	↑ MMSE score (*P* = .04) and WMS-LM I immediate recall (*P* = .03) in multidomain intervention group compared with control after 6 mo. Phonemic fluency not significant post hoc. Category fluency, DSST, and Stroop not significant.
			Active control: 25	76.8 (6.8)	44		MMSE: 26.6 (1.6)			
Thapa et al,^57^ 2020	South Korea	Community	Multidomain intervention: 34	72.6 (5.4)	82	Neurological examination and neuropsychological assessments by a dementia specialist.	MMSE: 26.0 (1.8)	Multidomain intervention: virtual reality–based cognitive training (eg, memory) and education program for 100 min, 3/wk for 8 wk, sequential. Active control: education program on general health (eg, nutrition, exercise) for 30-50 min, 1/wk for 8 wk. Interventions supervised in-person by health professionals, an exercise specialist, a physical therapist, and a nutritionist.	Baseline, 8 wk.	↑ TMT-B performance in multidomain intervention group compared with control (*P* = .03). No differences in MMSE, TMT-A, and DSST.
			Active control: 34	72.7 (5.6)	71		MMSE: 26.3 (3.3)			
Zheng et al,^58^ 2021	China	Community	Multidomain exercise: 23	65.8 (4.4)	74	Petersen criteria; MoCA score <26.	MoCA:22.3 (2.4)	Multidomain exercise: Baduanjin exercise and health education program for 60 min, 3/wk for 24 wk, sequential. Multidomain walk: brisk walking and health education program for 60 min, 3/wk for 24 wk. Active control: health education program 30 min, 1 every 8 wk. Intervention supervised in-person by professional coaches.	Baseline, 24 wk.	↑ MoCA (*P* = .005), WMS composite (*P* = .008), and WMS subdomains of memory quotient (*P* = .01), mental control (*P* = .02), comprehension memory (*P* = .008) scores at 6 mo in multidomain group compared with control.
			Multidomain walk: 23	64.9 (3.3)	52		MoCA: 21.7 (2.4)			
			Active control: 23	65.9 (5.3)	74		MoCA: 20.8 (3.3)			

Abbreviations: ↑, greater improvement; ADAS-cog, Alzheimer Disease Assessment Scale–Cognitive Subscale; aMCI, amnestic mild cognitive impairment; AVLT, auditory verbal learning test; BADL, Bayer-Instrumental Activities of Daily Living; CDR, Clinical Dementia Rating scale; CDR-SOB, Clinical Dementia Rating Scale Sum of Boxes; DHA, docosahexaenoic acid; DRT, disjunctive reaction time; DSST, Digit Symbol Substitution Test; FA, fatty acid; FAB, Frontal Assessment Battery; *ICD-9-CM*, International Classification of Diseases, 9th Revision, Clinical Modification; MCI, mild cognitive impairment; MMSE, Mini-Mental State Examination; MoCA, Montreal Cognitive Assessment; naMCI, nonamnestic MCI; NCGG-FAT, National Center for Geriatrics and Gerontology functional assessment tool; NR, not reported; RAVLT, Rey Auditory Verbal Learning Test; RBANS, Repeatable Battery for the Assessment of Neuropsychological Status; tDCS, transcranial direct current stimulation; TMT, Trail Making Test; WAIS, Wechsler Adult Intelligence Scale; WMS-LM, Wechsler Memory Scale Logical Memory; WTAR, Wechsler Test of Adult Reading.

^a^ Sex not reported.

^b^ Data for the whole sample.

^c^ Mean age not reported.

### Multidomain Intervention Characteristics

Multidomain interventions included cognitive and physical components^35,36,37,39,41,42,43,44,45,48,52^; multiple exercise^33,40,49,50,55,56^ or cognitive components^34^; nutritional supplements^33,47^; mind-body^38,46,54^; education^57,58^; cognitive, physical, and social components^31^; combined cognitive training with transcranial direct current stimulation (tDCS)^51^; and exercise with music (Table 1).^53^ Eighteen studies conducted the intervention components sequentially,^31,33,34,36,37,38,40,41,42,43,44,46,47,48,49,54,57,58^ whereas 10 studies conducted them simultaneously.^32,35,39,45,50,51,52,53,55,56^ Interventions were also completed in a group setting in 19 studies^31,33,34,35,36,38,39,40,45,47,48,49,50,52,53,54,55,56,58^ compared with the remaining 9, which were conducted individually.^32,37,41,42,43,44,46,51,57^ Lastly, the active control in 21 studies contained 1 component of the multidomain intervention,^32,34,35,36,37,38,39,40,41,42,43,44,45,46,47,48,51,53,54,57,58^ whereas 7 studies used alternative interventions.^31,33,49,50,52,55,56^ Cognitive outcomes converged under 7 domains: attention, executive function, global cognition, memory, processing speed, verbal fluency, and other (eg, reaction time, visuospatial) (Table 2).

**Table 2.** Summary of Cognitive Outcomes by Cognitive Domain

Source	Attention	Executive function	Global cognition	Memory	Processing speed	Verbal fluency	Other
Bae et al,^31^ 2019	NA	= TMT-A; = TMT-B	= MMSE	↑ Corsi block-tapping task; = Immediate and delayed recall composite score	= DSST	NA	NA
Bai et al,^32^ 2021	NA	= Digit span	NA	NA	NA	NA	WAIS subtest: ↑ Arithmetic, block design, picture arrangement. = Information, picture completion.
Bisbe et al,^33^ 2020	NA	= TMT-A; = TMT-B	= MMSE	↓ WMS-LM III; = RBANS	NA	↑ Category; = Letter	Visuospatial: = JLO
Buschert et al,^34^ 2011	NA	= TMT-A; = TMT-B	↑ ADAS-Cog; = MMSE	= RBANS	NA	NA	NA
Combourieu Donnezan et al,^35^ 2018	NA	↑ Digit Span Forward; ↑ Digit Span Backward; ↑ Matrix Reasoning; = Stroop Color Word	NA	NA	NA	NA	NA
Damirchi et al,^36^ 2018	NA	= Forward digit span	NA	NA	= DSST	NA	= Stroop errors and reaction time
Fiatarone Singh et al,^37^ 2014	NA	= Composite score (WAIS-III, semantic and phonemic verbal fluency)	↓ ADAS-Cog	= Composite score (ADAS-Cog, BVRT, WMS-LM I/II)	= SDMT	↓ Category; = Letter: COWAT	NA
Fogarty et al,^38^ 2016	= TEA	= Digit Span; = TMT-A; = TMT-B;	NA	= HVLT; = RBMT–II	= DSST	NA	NA
Gill et al,^39^ 2016	NA	= TMT-A; = TMT-B	↑ Composite score	↑ AVLT	= DSST	↑ Category: Animal naming; ↑ Letter: COWAT	NA
Greblo Jurakic et al,^40^ 2017	= MoCA attention subtest	↑ MoCA visuospatial/executive subtest	↑ MoCA	= MoCA delayed recall subtest	NA	= MoCA language subtest	↑ MoCA orientation subtest
Hagovská et al,^41^ 2016	NA	= TMT-A	NA	NA	NA	NA	NA
Hagovská et al,^42^ 2016	↑ ACE subtest	NA	↑ ACE composite score	↑ ACE subtest	NA	↑ ACE subtest	Visuospatial: = ACE subtest
Hagovská et al,^43^ 2016	NA	NA	↑ MMSE	NA	NA	NA	NA
Hagovská et al,^44^ 2016	NA	↑ Stroop Color-Word; ↑ TMT-A	↑ MMSE	↑ AVLT	↑ DRT-II	NA	NA
Jeong et al,^45^ 2021	NA	↑ TMT-A; ↑ TMT-B	= ADAS-Cog; = MMSE	NA	↑ DSST	NA	NA
Kim et al,^46^ 2020	NA	NA	= ADAS-Cog; = MoCA	NA	NA	NA	NA
Köbe et al,^47^ 2016	= Composite score	= Composite score	NA	= Composite score	NA	NA	Sensorimotor: = Composite score
Lam et al,^48^ 2015	NA	= Digit Span Forward; = Digit Span Backward; = TMT-A; = TMT-B	= CDR-SOB; = MMSE	= Delayed recall ADAS-cog subtest	NA	= Category	NA
Li et al,^49^ 2021	↑ MoCA subtest	↑ MoCA subtest	↑ MMSE; ↑ MoCA	↑ MoCA subtest	NA	↑ MoCA subtest	↑ MoCA abstraction and orientation subtests
Makizako et al,^50^ 2012	NA	NA	NA	NA	NA	NA	= Reaction time (handheld button); = Dual task costs
Martin et al,^51^ 2019	= RVIP CANTAB subtest	NA	NA	= CVLT; = PAL CANTAB subtest	= DSST	NA	NA
Shimada et al,^52^ 2018	NA	= TMT completion	↑ MMSE	↑ WMS-LM II; = RAVLT	NA	↑ Category; ↑ Letter	NA
Shimizu et al,^53^ 2018	NA	NA	= FAB	NA	NA	NA	NA
Sun et al,^54^ 2021	NA	NA	↑ MMSE; ↑ MoCA	NA	NA	NA	NA
Suzuki et al,^55^ 2013	NA	NA	= ADAS-Cog; ↑ MMSE	↑ WMS-LM I; = WMS-LM II	NA	NA	NA
Suzuki et al,^56^ 2012	NA	= Stroop Color-Word	↑ MMSE	↑ WMS-LM I; = WMS-LM II	= DSST	= Category; = Letter	NA
Thapa et al,^57^ 2020	NA	= TMT-A; ↑ TMT-B	= MMSE	NA	= DSST	NA	NA
Zheng et al,^58^ 2021	NA	WMS subtests: = Digit span; ↑ Mental control	↑ MoCA	↑ WMS composite; WMS subtests: = Memory quotient; = Picture recall and recognition; ↑ Picture reproduction; ↑ Comprehension memory	NA	NA	NA

Abbreviations: =, no difference; ↑, greater improvement in intervention group; ↓, greater improvement in control group; ACE, Addenbrooke’s Cognitive Examination; ADAS-Cog, Alzheimer Disease Assessment Scale-Cognitive subscale; AVLT, Auditory Verbal Learning Test; BVRT, Benton Visual Retention Test; CANTAB, Cambridge Neuropsychological Test Automated Battery; CDR-SOB, Clinical Dementia Rating Scale Sum of Boxes; COWAT, Controlled Oral Words Association Test; CVLT, California Verbal Learning Task; DRT-II, disjunctive reaction time; DSST, Digit Symbol Substitution Test; FAB, Frontal Assessment Battery; HVLT, Hopkins Verbal Learning Test; JLO, Judgment of Line Orientation; MoCA, Montreal Cognitive Assessment; MMSE, Mini-Mental State Examination; NA, not applicable; PAL, Paired Associates Learning; RAVLT, Rey Auditory Verbal Learning Test; RBANS, Repeatable Battery for the Assessment of Neuropsychological Status; RBMT–II, Rivermead Behavioral Memory Test, Second Edition; RVIP, Rapid Visual Information Processing; SDMT, Symbol Digit Modalities Test; TEA, Test of Everyday Attention; TMT, Trail Making Test; WAIS, Wechsler Adult Intelligence Scale; WMS-LM, Wechsler Memory Scale–Logical Memory.

### Associations of Multidomain Interventions With Cognitive Outcomes

#### Global Cognition

**Figure 2.**
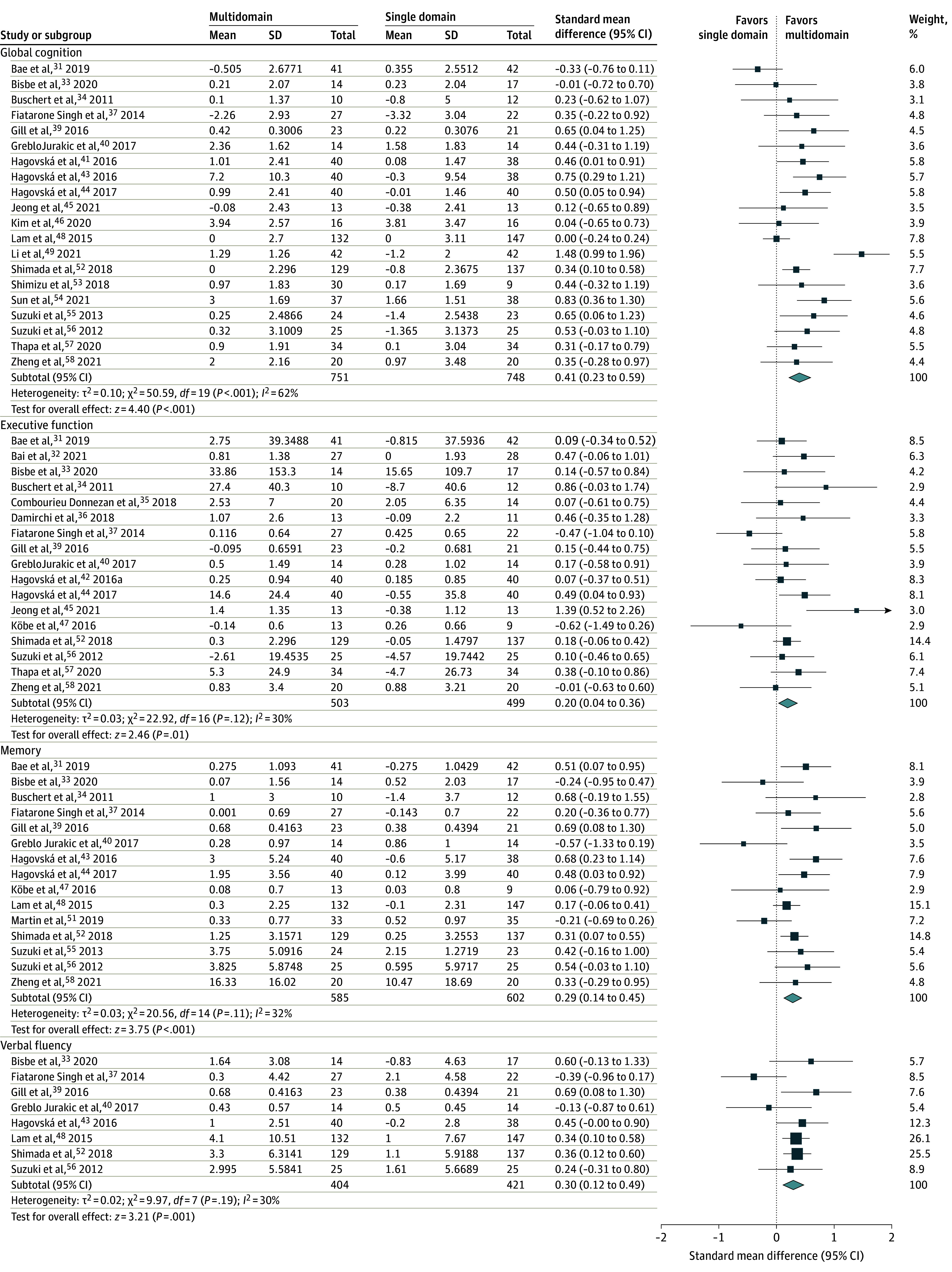
Forest Plots by Cognitive Domain Horizontal lines indicate the 95% CIs. The size of the square data marker refers to the proportional weight of each study. The diamond represents the pooled effect size.

Twenty studies evaluated global cognition, of which 12 reported improvements^34,39,40,42,43,44,49,52,54,55,56,58^ and 7 found no differences in the multidomain intervention compared with the control (Table 2).^31,33,45,46,48,53,57^ One study reported increased global cognition scores in the multidomain group, but the changes were smaller than the control.^37^ The pooled effect size favored the multidomain intervention (SMD, 0.41; 95% CI, 0.23-0.59; *P* < .001) such that participants in the multidomain group had improved global cognition immediately after the intervention compared with those in the single-domain control (Figure 2A). A medium level of heterogeneity (*I^2^* = 62%) was present, leading to stratification of the sample by recruitment source, order of multidomain intervention components, multidomain and control group intervention styles, and multidomain intervention type, but there were no differences between the subgroups.

**Figure 3.**
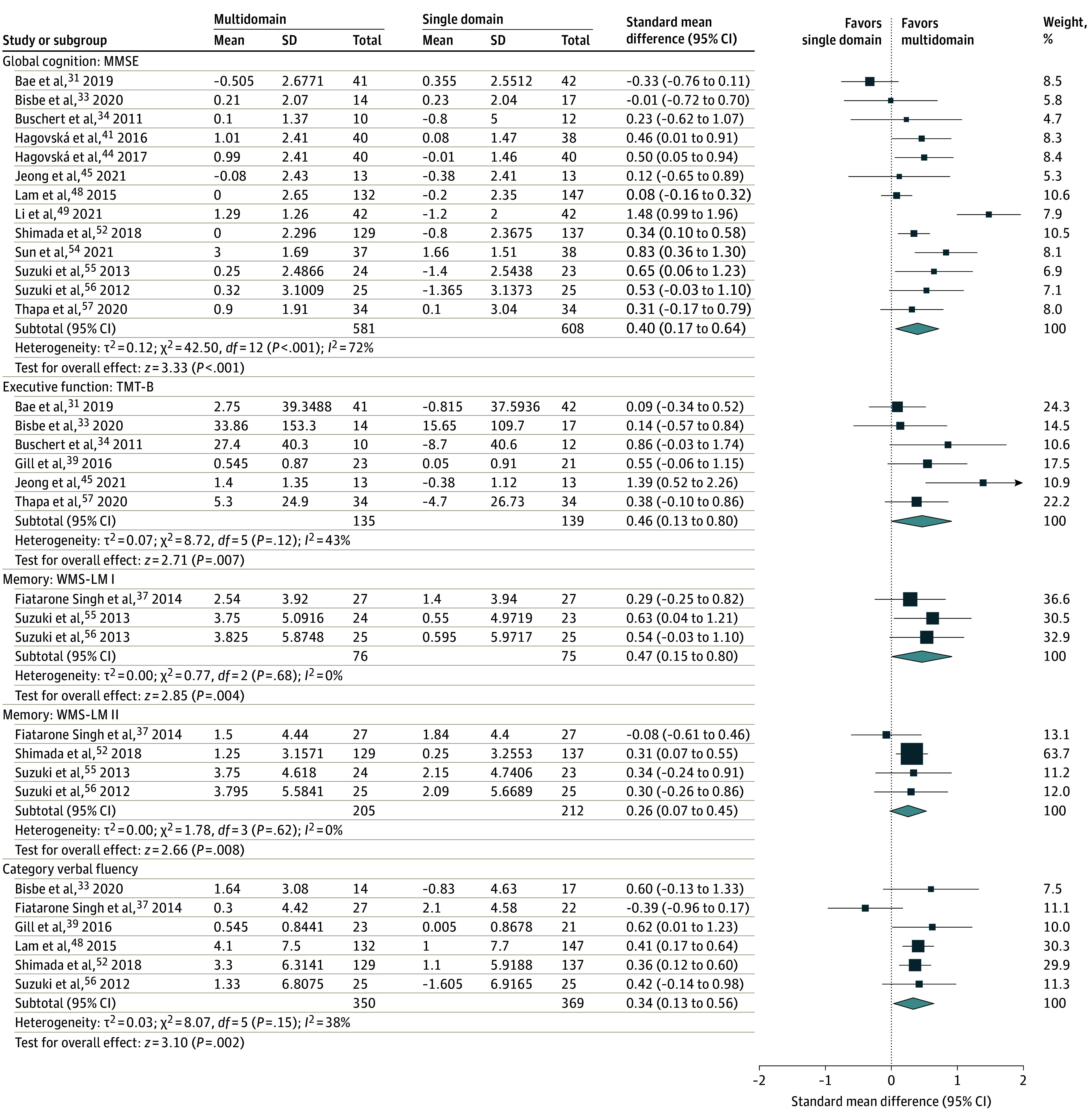
Forest Plots of Cognitive Tests Horizontal lines indicate the 95% CIs. The size of the square data marker refers to the proportional weight of each study. The diamond represents the pooled effect size. MMSE indicates Mini-Mental State Examination; TMT-B, Trail Making Test, Part B; and WMS-LM, Wechsler Memory Scale–Logical Memory.

Five studies measured cognition using the Alzheimer Disease Assessment Scale–Cognitive Subscale (ADAS-Cog), but cognitive outcomes were not significantly different between multidomain and control groups. Thirteen studies using the MMSE demonstrated that the overall pooled effect size favored the multidomain intervention (SMD, 0.40; 95% CI, 0.17-0.64; *P* < .001) such that there was a greater increase in MMSE scores after the intervention in the multidomain group compared with the control group (Figure 3A).^31,33,34,43,44,45,48,49,52,54,55,56,57^ Medium heterogeneity (*I^2^* = 72%) was present, but stratifying the studies by control group intervention type, recruitment source, and order of the multidomain intervention components found that subgroups were not significantly different.

#### Executive Function

Twenty studies evaluated executive function, but 3 studies^38,48,49^ were omitted from analyses due to missing data.^31,32,33,34,35,36,37,39,40,41,44,45,47,52,56,57,58^ Five studies found positive associations^44,45,49,57,58^ compared with 1 that reported a smaller effect size in the multidomain group compared with the control group.^37^ The remaining studies found no differences between intervention and control groups.^31,32,33,34,35,36,38,39,40,41,47,48,52,56^ Overall, the pooled effect size favored the multidomain intervention (SMD, 0.20; 95% CI, 0.04-0.36; *P* = .01) (Figure 2B). Medium heterogeneity (*I^2^* = 30%) was present, leading to stratification of the sample by recruitment source, order of multidomain intervention components, multidomain and control group intervention styles, and multidomain intervention type, but there were no differences between the subgroups.

By examining specific tests of executive function, 7 studies measured the Trail Making Test Part A^31,34,39,41,44,45,57^ and 4 studies measured the Stroop test,^35,36,44,56^ but there were no differences between groups. There was, however, a medium effect size across 6 studies that demonstrated significant improvements on the Trail Making Test Part B in the multidomain group (SMD, 0.46; 95% CI, 0.13-0.80; *P* = .007) with medium heterogeneity (*I^2^* = 43%). The small number of studies prevented further subgroup analyses (Figure 3B).^31,33,34,39,45,57^

#### Memory

Seventeen studies evaluated memory, whereby 15 were included^31,33,34,37,39,40,42,44,47,48,51,52,55,56,58^ in the meta-analyses because of incomplete data in 2 studies.^38,49^ Seven studies reported no differences,^34,37,38,40,47,48,51^ 1 reported greater improvements in the control group,^33^ and 9 reported greater changes in the multidomain group.^31,39,42,44,49,52,55,56,58^ This is supported by the pooled effect size; greater improvements in memory were observed immediately after the intervention in the multidomain group compared with the control group (SMD, 0.29; 95% CI, 0.14-0.45; *P* < .001) (Figure 2C). Given medium heterogeneity (*I^2^* = 32%), studies were stratified according to recruitment source, multidomain and single intervention type, multidomain intervention style, and intervention order, but there were no differences between subgroups.

The Wechsler Memory Scale Logical Memory I (WMS-LM I; SMD, 0.47; 95% CI, 0.15-0.80; *P* < .001) and II (WMS-LM II; SMD, 0.26; 95% CI, 0.07-0.45; *P* < .001) favored greater improvements in memory in the multidomain intervention group compared with the control group (Figure 3C and D). An insufficient number of studies prevented subgroup analyses.

#### Verbal Fluency

Nine studies evaluated verbal fluency, whereby 1 study was excluded from analyses due to missing data.^49^ Five studies found a positive association in the multidomain intervention group^33,39,42,49,52^ compared with 3 that revealed no differences^40,48,56^ and 1 study that found greater changes in the control group.^37^ The overall effect size favored improvements in the multidomain intervention group (SMD, 0.30; 95% CI, 0.12-0.49; *P* = .001) but with medium heterogeneity (*I^2^* = 30%) (Figure 2D). The studies were further subdivided based on the order of the multidomain invention components and the multidomain and single intervention type, but there were no differences between subgroups. In addition, 6 studies used category verbal fluency tests, which favored the multidomain group (SMD, 0.34; 95% CI, 0.13-0.56; *P* = .002) (Figure 3E). A medium level of heterogeneity was identified (*I^2^* = 38%), but there were no differences based on the order of interventions and single intervention type.

#### Attention

Six studies measured attention, but there were no differences between the multidomain intervention and control group in 5 studies^38,40,47,49,51^ compared with 1 that found improvements in the multidomain group.^44^ The overall effect size was, therefore, not significantly different between groups (SMD, 0.13; 95% CI, −0.15 to 0.41; *P* = .36).

#### Processing Speed

Ten studies evaluated processing speed, whereby 2 found improvements in the multidomain group^44,45^ and the remaining studies did not find differences between groups.^31,35,36,37,38,51,56,57^ Therefore, there were no discernable improvements after the intervention (SMD, 0.46; 95% CI, −0.04 to 0.96, *P* = .07).

#### Other Cognitive Domains

Eight studies targeted other cognitive domains, such as visuospatial, reaction time, and sensorimotor tests.^32,33,36,40,42,47,49,50^ Differences favored the multidomain group on the Wechsler Adult Intelligence Scale arithmetic, block design, and picture arrangement subtests, and MoCA abstraction and orientation subtests (Table 2).

### Exposure and Adherence to the Intervention

The multidomain interventions varied in terms of session duration (30-135 minutes), frequency (1-7 times per week), and length (8-52 weeks) (Table 2). The mean (SD) duration was 71.3 (36.0) minutes for 19.8 (14.6) weeks with sessions taking place 2.5 (1.1) times per week, and all interventions lasted less than a year. There were no adverse events related to the multidomain intervention, and adherence remained greater than 80% across all studies, with no differences between single and multidomain groups.

### RoB

The Egger test revealed that attention, executive function, global cognition, memory, processing speed, and verbal fluency funnel plots were symmetrical, indicating minimal risk of publication bias (eFigure 1 in the Supplement). A summary of all RoB criteria using Cochrane’s RoB tool is presented in eFigure 2 in the Supplement. In particular, the blinding of participants and personnel proved to be the most variable, whereby 9 studies presented a low risk of bias,^31,32,33,37,39,48,51,52,55^ 9 studies demonstrated a high risk of bias,^34,41,42,43,44,46,49,54,58^ and 10 studies were unclear.^35,36,38,40,45,47,50,53,56,57^ However, this factor is unlikely to have affected the outcomes of this analysis.

## Discussion

### Associations of Multidomain Interventions With Outcomes

The purpose of this systematic review and meta-analysis was to evaluate whether multidomain interventions were associated with greater improvements in cognition than single interventions in older adults with MCI. Findings from this review predominantly featured multidomain cognitive-physical interventions, which reflects previously reported interventions in the literature.^10,17^ However, nutrition, mind-body, music, and social interventions also contributed to small-medium effect sizes in global cognition, executive function, memory, and verbal fluency immediately after the intervention. Attention and processing speed did not differ between intervention groups, and an insufficient number of studies prevented pooling other cognitive outcomes, such as reaction time and visuospatial abilities.

Because of medium heterogeneity, subgroup analyses were conducted, but differences were not found across recruitment source, multidomain intervention type, single intervention type, multidomain intervention style, and order of the multidomain intervention components, which may be because of the limited number of studies in each subgroup. Nonetheless, these differences should be considered when designing new interventions, as they contributed to methodological variability. For example, diagnosis of MCI may lack uniformity among participants recruited from the community compared with a memory clinic.^59^ In addition, patients from a clinic may be at a greater risk of progressing to dementia.^60^ There is also mixed evidence on the benefits of sequential vs simultaneous cognitive-physical training interventions.^10,16,61^

A second analysis was conducted to determine whether specific cognitive tests acted as a potential source of heterogeneity between studies. Findings revealed improved scores on the MMSE in the global cognition domain, TMT-B in the executive function domain, category verbal fluency scores, and WMS-LM I and II in the memory domain that significantly favored the multidomain intervention group compared with the single-intervention control group. Although the effect sizes for these findings were small to medium, cognitive changes at the predementia stage could be of clinical relevance and important for public health. In all cases, effect sizes in global cognition, executive function, memory, and verbal fluency scores favored the multidomain group. Therefore, changes occurring immediately after the intervention may be used to assess the effectiveness of multidomain training on global cognition and specific cognitive domains and has the potential to inform MCI prognosis. Previous meta-analyses examining the association of cognitive or physical training alone with cognition among patient with MCI have found small to medium effect sizes favoring the intervention group.^5,62^ Similar findings were reported in global cognition, memory, and executive function following multidomain interventions.^16,18,19^ The present meta-analysis further contributes to the existing literature by demonstrating similar interactions that are not limited to cognitive and physical interventions. Findings from this study, however, must be interpreted with an understanding that effect sizes do not indicate clinical significance on tests such as the MMSE, which have limited sensitivity to detect meaningful changes in MCI. Rather, effect sizes can help with the interpretation of changes occurring in different cognitive domains.

### Exposure and Adherence to the Multidomain Intervention

Compared with single interventions, synergistic benefits arising from multidomain interventions align with the multifactorial nature of MCI. It is important, however, to consider the reproducibility, scalability, and adherence to such interventions. Determining the optimal exposure to a multidomain intervention is uniquely challenging in that there is no gold standard intervention combination and the association of each intervention with improvements in cognition is best understood by comparing trials with multiple arms. While most studies compared 1 intervention component to the multidomain intervention, a study comparing both arms reported decreased cognitive test scores in the multidomain group, which contradicts the outcomes of the present meta-analysis.^37^ This may be attributed to stress and the demands of certain multidomain intervention combinations compared with others, which may lead to decreased benefits. More evidence is needed from multigroup trials to better understand the balance between demands and benefits of multidomain interventions.

All interventions were short term and lasted less than a year, but the number of sessions, duration, and length of intervention proved to be variable between studies. The rationale for choosing a specific intervention length was often not discussed but was likely derived from the standards for single interventions. Previous reports in the literature focusing on combined cognitive-physical interventions have suggested interventions ranging between 1 to 3 hours per week for at least 16 weeks and as long as 6 months, which is consistent with the studies presented in this analysis.^13,62^ The effectiveness and length of exposure to more novel interventions, such as mind-body or tDCS, have not been examined to the same extent as cognitive-physical interventions.^31,47,53^ Future studies should continue investigating these interventions, as improved cognitive outcomes favored the multidomain group.

Studies reported high adherence to the multidomain intervention, which was similar between multidomain and active control groups. This contradicts previous reports suggesting that only one-third of older adults adhere to recommendations of single interventions, such as physical activity.^63^ Adherence to multidomain interventions was also found in a longer-term, 2-year trial that reported improved scores on a comprehensive neuropsychological test battery.^64^ Therefore, multidomain interventions remain engaging and should be contrasted with single domain interventions to examine differences in adherence during longer-term trials. Finally, there were no reports of adverse events related to the multidomain interventions, indicating their safety for older adults with MCI.

### Limitations

This study has limitations need to be acknowledged. First, distinct cognitive outcomes were assessed in studies from the same research groups, but bias may be introduced if participants were corecruited for these studies.^41,42,43,44,55,56^ To minimize reporting bias, data extraction and study outcomes were discussed among 3 independent reviewers. Additionally, the selected studies lacked sufficient data to account for different MCI subtypes (eg, amnestic MCI). Variability among different types of multidomain intervention restricted direct comparisons between novel interventions, such as music and nutrition, and more commonly reported cognitive and physical interventions. Additionally, the control groups were composed of either a single component of the multidomain intervention or an alternative intervention, such as a health education program. In both cases, however, improvements in cognition remained significant in the multidomain group compared with the single intervention control, demonstrating the advantage of combining interventions in MCI.

## Conclusions

In this study, nonpharmacological, multidomain interventions mainly focused on physical exercise, cognitive training, mind-body, music, dietary supplements, social engagement, and education were associated with small to medium effect sizes indicating improvements in global cognition, executive function, memory, and verbal fluency. A synergistic association was found, suggesting combined interventions may be superior to single interventions to improve cognitive functioning in older adults with MCI. Furthermore, the MMSE, category fluency, TMT-B and WMS-LM scores improved in the multidomain group compared with the active control. Future studies should consider examining interventions other than combined cognitive-physical training, such as mindfulness and nutrition, as promising outcomes were found across a variety of novel interventions. More evidence is needed to determine the optimal exposure to the multidomain intervention and whether improvements are sustained longitudinally.

## Notes

Supplement. **eTable.** Database Search Strategy **eFigure 1.** Funnel Plot for Attention, Executive Function, Global Cognition, Memory, Processing Speed, and Verbal Fluency **eFigure 2.** Summary of RoB Criteria Using Cochrane’s RoB Tool
