# Supplementary material for: Associations of Multidomain Interventions With Improvements in Cognition in Mild Cognitive Impairment: A Systematic Review and Meta-analysis
Source: JAMA Netw Open. 2022 May 3;5(5):e226744. doi: 10.1001/jamanetworkopen.2022.6744 (PMC9066287; doi:10.1001/jamanetworkopen.2022.6744)
Supplement: Supplement. — eTable. Database Search Strategy eFigure 1. Funnel Plot for Attention, Executive Function, Global Cognition, Memory, Processing Speed, and Verbal Fluency eFigure 2. Summary of RoB Criteria Using Cochrane’s RoB Tool [file jamanetwopen-e226744-s001.pdf]

Supplemental Online Content

Salzman T, Sarquis-Adamson Y, Son S, Montero-Odasso M, Fraser S. Associations of multidomain interventions with improvements in cognition in mild cognitive impairment: a systematic review and meta-analysis. *JAMA Netw Open.* 2022;5(5):e226744. doi:10.1001/jamanetworkopen.2022.6744

eTable. Database Search Strategy

eFigure 1. Funnel Plot for Attention, Executive Function, Global Cognition, Memory, Processing Speed, and Verbal Fluency

eFigure 2. Summary of RoB Criteria Using Cochrane's RoB Tool

This supplemental material has been provided by the authors to give readers additional information about their work.

© 2022 Salzman T et al. *JAMA Network Open.*

eTable 1. Database Search Strategy

1	Cognitive dysfunction/
2	(mild cogniti* adj3 (dysfunction* or disorder* or impair* or declin* or deteriorat* or decrement*)).ti,ab,kf.
3	1 or 2
4	exp Aged/
5	(geriatric* or gerontolog* or elder* or senior* or retire*).ti,ab,kf.
6	((old* or aged) adj2 (individual? or person? or people or population? or adult? or patient? or group? or aged)).ti,ab,kf.
7	4 or 5 or 6
8	((combin* or two or dual* or multi* or comprehensiv*) adj5 (intervention* or train* or modal* or program* or therap* or treatment* or model* or rehabilitat* or program* or task* or exercis*)).ti,ab,kf.
9	((cognitive* or cognition* or brain* or mind* or spiritual* or mental* or psychology*) adj3 (motor* or physical* or body or bodies)).ti,ab,kf.
10	8 or 9
11[a]	(randomized controlled trial or controlled clinical trial).pt. or randomized.ab. or placebo.ab. or clinical trials as topic.sh. or randomly.ab. or trial.ti.
12[a]	exp animals/ not humans.sh.
13	11 not 12
14	3 and 7 and 10 and 13

[a]Lefebvre C, Glanville J, Briscoe S, et al. Searching for and selecting studies. In: Higgins JPT, Thomas J, Chandler J, et al., eds. *Cochrane Handbook for Systematic Reviews of Interventions*. 1st ed. Wiley; 2019:67-107. doi:10.1002/9781119536604.ch4

© 2022 Salzman T et al. *JAMA Network Open.*

eFigure 1. Funnel Plot for Attention, Executive Function, Global Cognition, Memory, Processing Speed, and Verbal Fluency

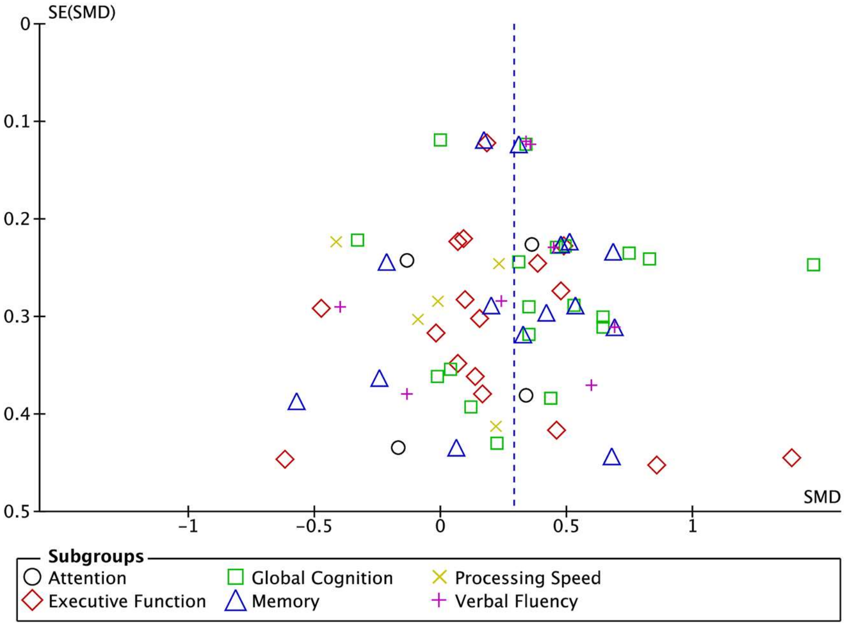

© 2022 Salzman T et al. *JAMA Network Open.*

eFigure 2. Summary of RoB Criteria Using Cochrane's RoB Tool

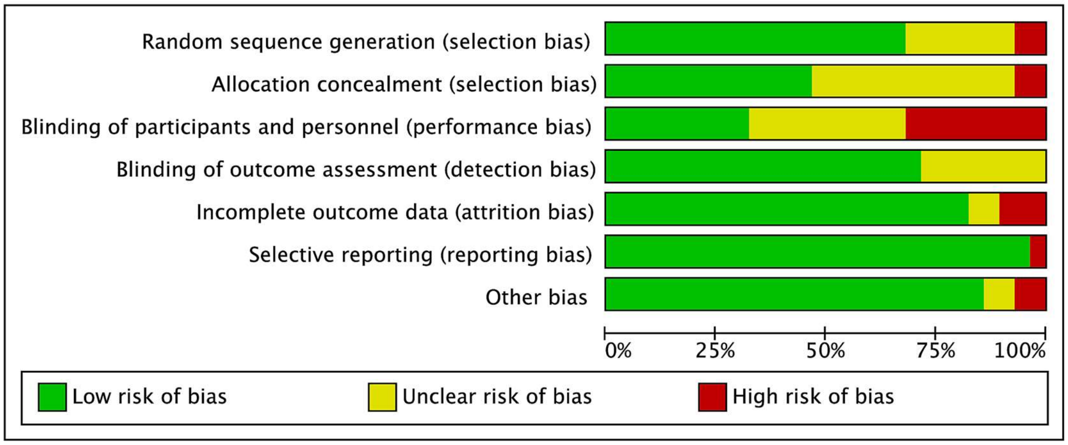

© 2022 Salzman T et al. *JAMA Network Open.*
